# Proton transfer-mediated GPCR activation

**DOI:** 10.1007/s13238-014-0106-4

**Published:** 2014-10-17

**Authors:** Xuejun C. Zhang, Can Cao, Ye Zhou, Yan Zhao

**Affiliations:** National Laboratory of Macromolecules, National Center of Protein Science—Beijing, Institute of Biophysics, Chinese Academy of Sciences, Beijing, 100101 China

**Keywords:** GPCR, activation, protonation, membrane potential

## Abstract

G-protein coupled receptors (GPCRs) play essential roles in signal transduction from the environment into the cell. While many structural features have been elucidated in great detail, a common functional mechanism on how the ligand-binding signal is converted into a conformational change on the cytoplasmic face resulting in subsequent activation of downstream effectors remain to be established. Based on available structural and functional data of the activation process in class-A GPCRs, we propose here that a change in protonation status, together with proton transfer via conserved structural elements located in the transmembrane region, are the key elements essential for signal transduction across the membrane.

## Introduction

G-protein coupled receptors (GPCRs) comprise the largest and most diversified family of signaling membrane proteins in eukaryotic cells (Rohrer and Kobilka, 1998). They are a major class of targets of therapeutic intervention (Insel et al., 2007). In response to ligand binding from the extracellular face, GPCRs change their conformations on the cytoplasmic face to affect downstream intracellular events (Rasmussen et al., 2011). Understanding the common mechanisms of agonist-induced GPCR activation will facilitate designs of various ligands that manipulate the properties GPCR proteins. Thus, studying the mechanism is of both theoretical as well as practical importance.

**Figure 1:**
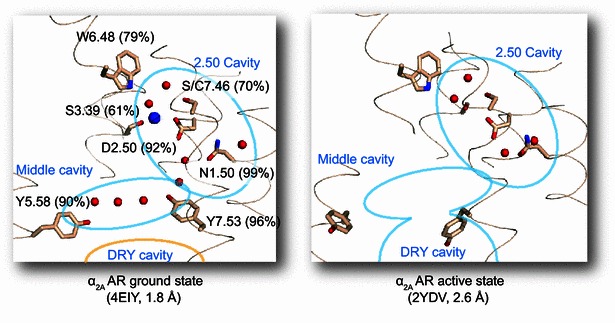
**Conserved proton wire in the major hydrogen-bond network of α**_**2a**_**AR**.  Key residues are shown as stick models, and water molecules and Na^+^ are shown in red and blue spheres, respectively. For clarity, only components along the major path of proton transfer are shown. Percentage conservativeness of the involved residues is included in parentheses. This figure is a modified version of a figure previously published by us (Zhang et al., 2013).

The majority of GPCRs (~85% (Tadevosyan et al., 2012)) belongs to the class-A subfamily. The structure of a class-A GPCR protein contains seven transmembrane (TM) helices (TMs 1–7) forming a TM core. Along the direction of the membrane normal, the TM core can be divided into three parts: The extracellular third is mainly responsible for ligand binding (Hanson and Stevens, 2009); the middle part is required for signal transduction (Zhang et al., 2013); and the intracellular third is responsible for interactions with downstream effectors (Rasmussen et al., 2011). To date, the precise function of the middle part remains enigmatic, yet this part contains most of the conserved structural elements of class-A GPCRs, including a major hydrogen-bond network (MHN) formed by the hydrophilic “2.50-cavity” and hydrophobic “middle-cavity” (Fig. 1) (Zhang et al., 2013; Katritch et al., 2013; Angel et al., 2009). These evolutionarily conserved elements are most likely to be the structural bases of a common, ligand independent, signal transduction mechanism of class-A GPCRs.

It has been proposed that interaction between protonated functional groups of the GPCR TM core and the ubiquitous, negative-inside, membrane potential plays a critical role in GPCR activation (Zhang et al., 2013). Open questions associated with such an activation mechanism include the following: What are the functional roles of conserved motifs in the activation mechanism? What is the status of GPCR protonation in its inactive (ground) state and in its active state? What do agonist and antagonist differ in terms of determining GPCR protonation? This mini-review addresses some of these questions, based mainly on the analysis of structural and functional data available for class-A GPCRs.

## Protonation status

GPCR activation is associated with both a conformational change from the ground state conformation (C_G_) to the active conformation (C_A_) and the opening of the cytoplasmic face of the protein (Rasmussen et al., 2011). For such a large change in overall structure to occur, energy in one form or another would be required. On the one hand, the binding energy of an agonist is usually small (∆G = RTln([L]/K_d_), where R represents the universal gas constant and T the temperature ~300K), especially when the ligand concentration is low in comparison with the dissociation constant (i.e. [L] ≈ K_d_), excluding this option as a viable energy source. On the other hand, under the influence of the electrostatic field of the evolutionarily conserved membrane potential (typically ∆Ψ ≈ −100 mV), proton movement from theextracellular side to the cytoplasmic side could potentially provide sufficient energy for the conformational change, provided proper coupling can be established (Zhang et al., 2013). For each proton to move across the membrane, this energy is F∆Ψ ≈ 4RT (where F is the Faraday constant). In general, when combined with the high-energy barrier of the transition state, extra energy input into the signaling process would increase the signal-to-noise ratio and thus permit faithful yet low-noise responses to a variety of ligands of a wide range of binding affinities.

Proton-titratable residues (or functional groups) are essential for such regulated proton transfer. For a typical class-A GPCR, there are only two conserved acidic residues embedded in the TM region, namely D2.50 (as per B–W numbering (Ballesteros and Weinstein, 1995)) in the conserved MHN and D/E3.49 of the DRY motif, and they are the most likely candidates for protonation sites (Zhang et al., 2013). Since D/E3.49 (conserved in 94% class-A GPCRs, as estimated according to the online database 7TM Alignment Explorer (Van Durme et al., 2006)) is located downstream of D2.50 (92%) in the electrostatic field, the initial protonation site is likely to be D2.50; and the final destination is likely to be D/E3.49. In between these two residues, multiple functional groups of the MHN may provide a proton-relaying framework, including a cluster of ordered water molecules which can be protonated as well (Fig. 1). As long as a conformational change allows movement of the proton along the electrostatic field, such a change would be favored in the presence of membrane potential. Thus, proton transfer from D2.50 to D/E3.49 may generate energy that favors the C_G_-to-C_A_ conformational change.

In all potential-bearing membranes, the equilibrium conformation of a charge-carrying membrane protein is the result of a balance between forces of electrostatic potential and a hydrophobic mismatch. The lipid bilayer functions like a belt around the structure of the GPCR protein. The location at which the belt interacts with a GPCR determines the conformation and functional status of the latter, including ligand potency and efficacy. From the protein point of view, in addition to non-specific hydrophobic interaction, a GPCR molecule is anchored within the lipid bilayer via a number of specific interactions, including the amphipathic helix-8 (H8), which fixes the position of TM7 relative to the membrane. These anchor positions may serve as pivots when the GPCR responds to external forces. Because of the different locations the two conserved acidic residues reside relative to these pivots, protonation events at D2.50 and D/E3.49 may result in distinct effects on GPCR conformation. In a more general sense, when a proton moves within the proton-relay path, different parts of the protein may experience forces and torques at different time points, a process that ultimately affects the dynamic properties of both the TM helices (including their positions and orientations relative to each other) and the overall conformation of the GPCR TM core as well.

## Activation of class-A GPCR

**Figure 2:**
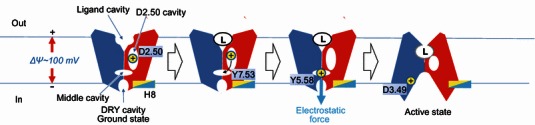
Schematic diagram of the putative GPCR activation process

On the basis of distinct micro-environments of D2.50 in the C_G_ and C_A_ states, we previously proposed a conformational switch that is centered around D2.50 (Zhang et al., 2013). In particular, a change in protonation status of D2.50 is the initial step in a cascade of conformational changes that results in the activation of the GPCR. The exact protonation status at D2.50, however, has remained an open question until now. Here, based on further structural analysis, we propose a mechanism by which the trigger for activation is not protonation of the GPCR *per se*, but the actual process of proton transfer from an inert position (D2.50) to an active position (the middle-cavity). This putative activation process will be described below (Fig. 2).

(i) D2.50 is protonated in the C_G_ state. This assumption on the protonation status of D2.50 is based on the structural observation that D2.50 is deeply embedded in the middle of the TM core as well as a need for a proton source at the beginning of the activation process. Mutations at D2.50 (including D2.50 N) in a number of GPCRs inevitably result in abolishing GPCR activity (Bihoreau et al., 1993; Parent et al., 1996; Proulx et al., 2008; Strader et al., 1988; Ceresa and Limbird, 1994; Martin et al., 1999), suggesting that the ability of this position to switch between protonated and deprotonated status is essential for GPCR activation. Furthermore, sodium ion (Na^+^) wasfound to bind to D2.50 in several crystal structures of GPCRs in their C_G_ states (Zhang et al., 2012; Liu et al., 2012), implying that D2.50 is able to attract positively charged ions. Thus, the absence of the Na^+^ very likely results in the protonation of D2.50 (or binding of a protonated water molecule H_3_O^+^). The bound proton most likely originates from the extracellular space, with its movement driven by the negative-inside membrane potential. The corresponding proton-binding energy may be utilized to put the GPCR into a permissive state ready for signaling. In the presence of a membrane potential, this protonation site is subjected to an electrostatic force, and this force can be balanced by the hydrophobic mismatch force established by the presence of the conserved amphipathic helix H8. In particular, since TMs 1, 2, and 7 form a rigid body (Zhang et al., 2013), the force applied onto D2.50 can be balanced by H8 via TM7.
(ii) Agonist binding induces a characteristic change in the micro-environment of D2.50, including an “upshift” (i.e. towards the extracellular space) of TM3 relative to D2.50, which brings the conserved S3.39 (61%) to the vicinity of D2.50 (Zhang et al., 2013). In response to the approaching movement of S3.39, deprotonation occurs at D2.50. Since most D2.50N mutations show lower agonist affinity and become less active in response to agonist (Wilson et al., 2001), a protonated D2.50 appears to be incompatible with agonist binding, thus deprotonation is favored. In addition, in bacteriorhodopsin (a 7-TM, photon-catalyzed, proton pump), the moving of a threonine residue (T46) closer to an acidic residue (D96), from 3.0 Å to 1.85 Å, is believed to reduce the pK_a_ of the latter by 5.5 units (Onufriev et al., 2003). This suggests that similar deprotonation may occur at D2.50 of the GPCR upon moving closer to the residue S3.39. In turn, this deprotonation event can stabilize the new conformation of S3.39 relative to D2.50.
(iii) The released proton is transferred from the 2.50-cavity to a linearly aligned cluster of three water molecules in the middle-cavity (Fig. 1). Subsequently, one of the water molecules gets protonated (i.e. forming H_3_O^+^). Because of the negative-inside membrane potential and the deeply embedded positon of D2.50, the released proton is unlikely to move to the extracellular space. Furthermore, the proton transfer requires Y7.53 (96%) of the NPxxY motif, which is located between the two cavities. Using its side chain hydroxyl group, a tyrosine residue may function as a proton-relay intermediate. Y7.53F mutation has been shown to reduce GPCR activation (e.g. in A_T2_R), an effect similar to that of the D2.50N mutation (Marie et al., 1994). Other parts of theproton-wire are formed by ordered water molecules, which are precisely positioned by polar residues and mainchain atoms from highly conserved motifs (Zhang et al., 2013). In a typical class-A GPCR, the middle-cavity is located downstream of D2.50 in the electrostatic field, thus allowing the proton transfer to be powered by the membrane potential.
(iv) Because the middle-cavity is formed mainly by conserved hydrophobic residues, including those from TM6 (Zhang et al., 2013), the protonated water molecule applies a force to the overall structure of the GPCR and TM6 in particular. The middle-cavity region was previously referred to as TM6-clamp (Hulme, 2013), implying its functional role in TM6 movement. Protonation of a buried water cluster has been shown to be important for functions of bacteriorhodopsin as well (Garczarek et al., 2005). In general, maintaining water molecules in a hydrophobic cavity inside a protein is energetically costly (Nucci et al., 2014), suggesting an important functional role of the middle-cavity. The hydrophobic interior of the middle-cavity provides insulation for the enclosed water cluster, separating it from the DRY pocket on the cytoplasmic side (Fig. 1). Such an insulation ensures both the provision of the protonation status of the water cluster and a focused electric field being applied on the protonated water molecule. Up to this point of the activation process, no major conformational change has occurred in the TM core region, except the relative movement between TMs 2 and 3 responsible for triggering the deprotonation of D2.50.
(v) In response to the “downward” (i.e. towards the cytoplasm) electrostatic force mediated by the protonated water molecule H_3_O^+^, TM6 moves away from the rest of the protein, resulting in the release of the water molecules from the middle-cavity, including that protonated. Being a characteristic feature of the GPCR activation (Rasmussen et al., 2011), this TM6 outward movement (i.e. away from the rest of the TM core; by about 14 Å at its cytoplasmic tip) enlarges the DRY-cavity, partially by merging it with the middle-cavity. This movement both deepens and widens the DRY-cavity, thus enabling its interaction with downstream G-proteins. This step may correspond to the change from the Meta I to Meta II state observed during rhodopsin activation, which is reported to associate with the release of hydrogen-bonded water molecules (Angel et al., 2009; Mitchell and Litman, 1999). The putative favorable effect of membrane potential on TM6 movement may explain the observation of an incomplete conformational change in many crystal structures of “active” GPCRs (Warne et al., 2011; White et al., 2012; Xu et al., 2011), since in these crystals the membrane potential is absent. *In vitro*, where there is neither a membrane potential nor a hydrophobic mismatch, a charge-carrying GPCR would be in an equilibrium conformation different from that observed under *in vivo*conditions.
(vi) Following the outward movement of TM6, a series of conformational changes occur in the DRY-cavity, as a consequence of which the C_A_ state is stabilized. By rearrangement of the inter-helix packing between TMs 3 and 6, R3.50 (98%) is released from the D/E3.49-R3.50 salt-bridge bond, which is a signature event in GPCR activation (Rasmussen et al., 2011; White et al., 2012; Palczewski et al., 2000). In the C_G_ state, D/E3.49 is deprotonated because of the proximate basic residue R3.50, while Y5.58 (90%) stabilizes the water cluster inside the middle-cavity. Upon the breaking of the salt-bridge, the pK_a_ of D/E3.49 rises, and D/E3.49 gets protonated. Meanwhile, R3.50 switches its partner, forming a hydrogen-bond with Y5.58 (White et al., 2012). This D/E3.49-protonation state corresponds to the Meta IIbH^+^ state of rhodopsin (Lohse et al., 2014). In addition, in *ca*. 38% of GPCRs, the position 2.39 is occupied by a threonine residue. This T2.39 is located in the vicinity of D/E3.49 in the C_G_ state (e.g. 2.7 Å in α2AAR/4EIY), but moves apart in the C_A_ state (e.g. 4.2 Å in α2AAR/2YDV). Similar to the above-mentioned distance change between S3.39 and D2.50, the positional shift of T2.39 away from D/E3.49 may promote and stabilize protonation of the latter. D/E3.49 may pick up a proton either directly from the proton-wire of the MHN or from the cytoplasm. As long as the pK_a_ of D/E3.49 is higher than the pH inside the DRY-cavity, protonation occurs spontaneously. In either case, the net result appears to be a proton transfer from D2.50 to D/E3.49. Since D/E3.49 is located at the end of the putative proton movement, a protonated D/E3.49 seems to stabilize the C_A_ state under the influence of membrane potential rather than to drive the conformational change.

## Additional issues related to protonation processes

In general, energy input would accelerate a molecular process in a particular direction, by overcoming energy barrier(s) of the transition-state(s). This is exemplified by the case of rhodopsin, where the energy input (~80RT at 600 nm wavelength) exceeds by far what is needed for the activation of a canonical class-A GPCRs (~4RT), namely by an order of magnitude. As a result, rhodopsin exhibits extremely high fidelity and reaction speed (Lohse et al., 2014). Furthermore, the kinetics of GPCR activation is usually much faster under *in vivo* conditions (~ 30 ms) than *in vitro* conditions (~30 s), indicating that factors critical for the conformational change during activation in the native environment are not available in purified or reconstituted systems (Lohse et al., 2014). In addition, in the presence of a membrane potential, the activation process is strongly favored in the forward direction. However, if deprotonation occurs (e.g. by dropping the proton into the cytoplasm at the end of the activation process), the equilibrium between the two states may shift. In principle, if the C_A_ state were unstable thermodynamically, a GPCR could function as a proton transporter by cycling between the C_G_ and C_A_ states. Interestingly, the rate of an inverse agonist-induced deactivation is usually slower than that of an agonist-induced activation (Lohse et al., 2014). This intriguing observation could be explained as follows: First, the favorable energy input, which is associated with the interaction between the proton and membrane potential in the C_G_-to-C_A_ activation process, is not available in the C_A_-to-C_G_ deactivation process. Second, an inverse agonist may achieve its effect through an alternative pathway (e.g. by re-loading a proton from the extracellular side) rather than a backward movement of the proton.

A smooth proton-transfer is essential for the full activation of GPCR. An inverse agonist may prevent deprotonation of D2.50, thus completely blocking activation. In contrast, basal activity of class-A GPCRs may originate from spontaneous deprotonation at D2.50. In addition, *in vivo* biased activation may be the result of different extents of the proton movement. For instance, if ligand binding only allows a half-way proton transfer, this may result in only a partial rather than full exposure of the effector binding site. Thus, ligand binding not only determines the direction but also the extent of the conformational change.

As mentioned above, in several high-resolution crystal structures of inactive GPCRs, Na^+^ is found to bind with D2.50 (Zhang et al., 2012; Liu et al., 2012). If a positive charge alone could trigger activation, Na^+^ binding would have a similar effect as protonation. However, effects of a positive charge on overall conformational change may be realized only when the charge moves along the electrostatic field of the membrane potential so that its electrostatic energy is converted to conformational energy of the protein. It should be noted that there is significant difference between protonation and Na^+^ binding. A proton may be easily transferred among titratable groups including water molecules, whereas Na^+^ may not. The MHN of class-A GPCR seems to be unsuitable for Na^+^ transfer (considering the elegant structural details of a Na^+^ channel (Payandeh et al., 2011)). In other words, the energy barrier for Na^+^ transfer appears much higher than that of a proton. In addition, no proper Na^+^-binding site is found in the C_A_ state. Thus, unlike protonation, Na^+^ seems unable to stabilize the C_A_ state. In fact, Na^+^ binding inhibits the activation of GPCR, probably by competing with protonation at D2.50 (Martin et al., 1999).

Interestingly, class-B GPCRs also contain conserved (class-specific) polar residues in their TM cores (Wootten et al., 2013; Hollenstein et al., 2013; Siu et al., 2013), including proton-titratable residues (e.g. the H2.50b-E3.50b pair (as per Wootten numbering (Wootten et al., 2013))). These polar residues are likely to interact with buried water molecules, and together they form a hydrogen-bond network. Mutations of most of these conserved polar residues result in impaired activity or reduced cell-surface expression of the receptors (Wootten et al., 2013). Most of these conserved polar residues are clustered into two groups, one in an orthosteric ligand-binding cavity facing the extracellular side, and the other in a cavity facing the cytoplasmic side. In addition, there is a small cavity in the vicinity of N5.50b, which binds an antagonist in the CRF_1_ receptor crystal structure (Hollenstein et al., 2013). Physiological agonists of class-B GPCRs are usually peptide hormones (Hollenstein et al., 2014). The N-terminus of the ligand peptide are thought to insert into the orthosteric binding cavity, although alternative binding modes are also proposed (Beinborn, 2006). In parallel to what we propose for activation of class-A GPCRs, it is probable that, in class-B GPCRs, the ligand binding couples with deprotonation in the orthosteric ligand-binding cavity (e.g. at the conserved H6.52), and the released proton transfers into the N5.50b cavity (functionally similar to the middle cavity in class-A GPCRs), triggering the opening of the cytoplasmic side. The final destination of the proton is likely to be the H2.50b-E3.50b pair located in the cytoplasmic cavity of this class of GPCRs.

## Conclusion Remarks

A fundamental question in GPCR activation concerns the mechanism that couples agonist binding at the extracellular side with the conformational opening at the cytoplasmic face. We hypothesize that it is the dynamics of protonation, rather than protonation *per se*, that affects the activation status of the class-A GPCR. During the activation process, a proton is translocated under the influence of membrane potential. In response, different parts of the GPCR experience forces at consecutive time points, determining the dynamics and kinetics of the activation process. Conserved motifs of class-A GPCRs, including D2.50, DRY, and NPxxY motifs, as well as a number of associated water molecules play essential roles in structure and functions of the proton-relay path.
